# Disjunct distribution and distinct intraspecific diversification of *Eothenomys melanogaster* in South China

**DOI:** 10.1186/s12862-018-1168-3

**Published:** 2018-04-10

**Authors:** Xue Lv, Jilong Cheng, Yang Meng, Yongbin Chang, Lin Xia, Zhixin Wen, Deyan Ge, Shaoying Liu, Qisen Yang

**Affiliations:** 1 Key Laboratory of Zoological Systematics and Evolution, Institute of Zoology, Chinese Academy of Sciences, 1 Beichen West Road, Chaoyang District, Beijing, 100101 People’s Republic of China; 2 College of Life Sciences, University of Chinese Academy of Sciences, Beijing, 100049 People’s Republic of China; 3 College of Life Sciences, Sichuan University, Chengdu, 610064 Sichuan People’s Republic of China; 4 Sichuan Academy of Forestry, No. 18, Xinghui Xilu Road, Chengdu, 610081 Sichuan People’s Republic of China

**Keywords:** Disjunct distribution, Distinct intraspecific diversification, *Eothenomys melanogaster*, Genetic divergence, Isolation by distance, Isolation by environment, Sky islands, South China

## Abstract

**Background:**

South China encompasses complex and diverse landforms, giving rise to high biological diversity and endemism from the Hengduan Mountains to Taiwan Island. Many species are widely distributed across South China with similar disjunct distribution patterns. To explore the causes of these disjunct distribution patterns and their genetic consequences, we investigated the endemic species Père David’s Chinese Vole (*Eothenomys melanogaster*) by integrating geological and ecological factors. We analysed the genetic structure and divergence time of *E. melanogaster* based on fast-evolving mitochondrial and nuclear markers using Bayesian trees and coalescent species tree approaches. Historical scenarios of distribution range and demography were reconstructed based on spatial interpolations of genetic diversity and distance, extended Bayesian skyline plots, phylogeographic diffusion analysis, and ecological niche modelling (ENM) during different periods. We also assessed the relationships between geographical distance/ecological vicariance and genetic distance (isolation by distance, IBD; isolation by environment, IBE).

**Results:**

The genetic analysis revealed three deeply divergent clades—Southeast, Southwest and Central clades, centred on the Wuyi Mountains, the Yunnan-Guizhou Plateau (YGP) and the mountains around the Sichuan Basin, respectively—that have mostly developed since the Pleistocene. IBD played an important role in early divergence, and geological events (sedimentation of plains and linking of palaeo-rivers) and IBE further reinforced genetic differentiation. ENM shows the importance of suitable habitats and elevations.

**Conclusions:**

Our results suggest that the primary cause of the disjunct distribution in *E. melanogaster* is the high dependence on middle-high-altitude habitat in the current period. Mountains in the occurence range have served as “sky islands” for *E. melanogaster* and hindered gene flow. Pleistocene climatic cycles facilitated genetic admixture in cold periods and genetic diversification in warm periods for inland clades. During cold periods, these cycles led to multiple colonization events between the mainland and Taiwan and erased genetic differentiation.

**Electronic supplementary material:**

The online version of this article (10.1186/s12862-018-1168-3) contains supplementary material, which is available to authorized users.

## Background

South China encompasses complex and diverse landforms. The high-altitude Hengduan Mountains (HM) and Yunnan-Guizhou Plateau (YGP) are in the west, and the terrain gradually descends to hilly areas in the east. The HM and adjacent areas are characterized by a series of parallel alpine ridges with dramatic ecological stratification and environmental heterogeneity [1, 2]. This complex topography supports one of the major biodiversity centres in the world, the southwest mountains of China [3, 4]. The mountain regions function as “sky islands” for montane species that depend exclusively on high-elevation environments [5, 6]. Dispersal of these species is strongly limited by low-elevation habitats and consequently results in genetic drifts among sky islands [7]. The southeast hilly areas, such as the Wuyi Mountains and Taiwan Island, are also described as hotspots for speciation and have high degrees of endemism [8, 9]. These regions have been influenced by tremendous climatic changes, with long-term cooler and drier tendencies during the Late Cenozoic [10, 11], and the uplift of the Qinghai-Tibetan Plateau (QTP).

The intraspecific diversification of montane species of South China is related to the uplift of the QTP since the Pliocene and the topographical complexity of the hilly areas [9, 12]. Previous studies have found that montane species migrate to low-elevation areas during glacial periods and exchange genes [13, 14]. Therefore, climatic fluctuations may also play an important role in shaping the genetic structure of montane species [8, 9]. Many species are widely distributed across South China [12], but montane species dwell at high elevations and are discontinuously distributed in this area [15, 16]. Disjunct distributions are very common in this area [9, 16]; however, in contrast to the extensively studied Southwest China, few studies have focused on the entirety of South China, particularly regarding the geographical and ecological vicariance of species within their geographical ranges and their geographical patterns of genetic diversity.

**Fig. 1:**
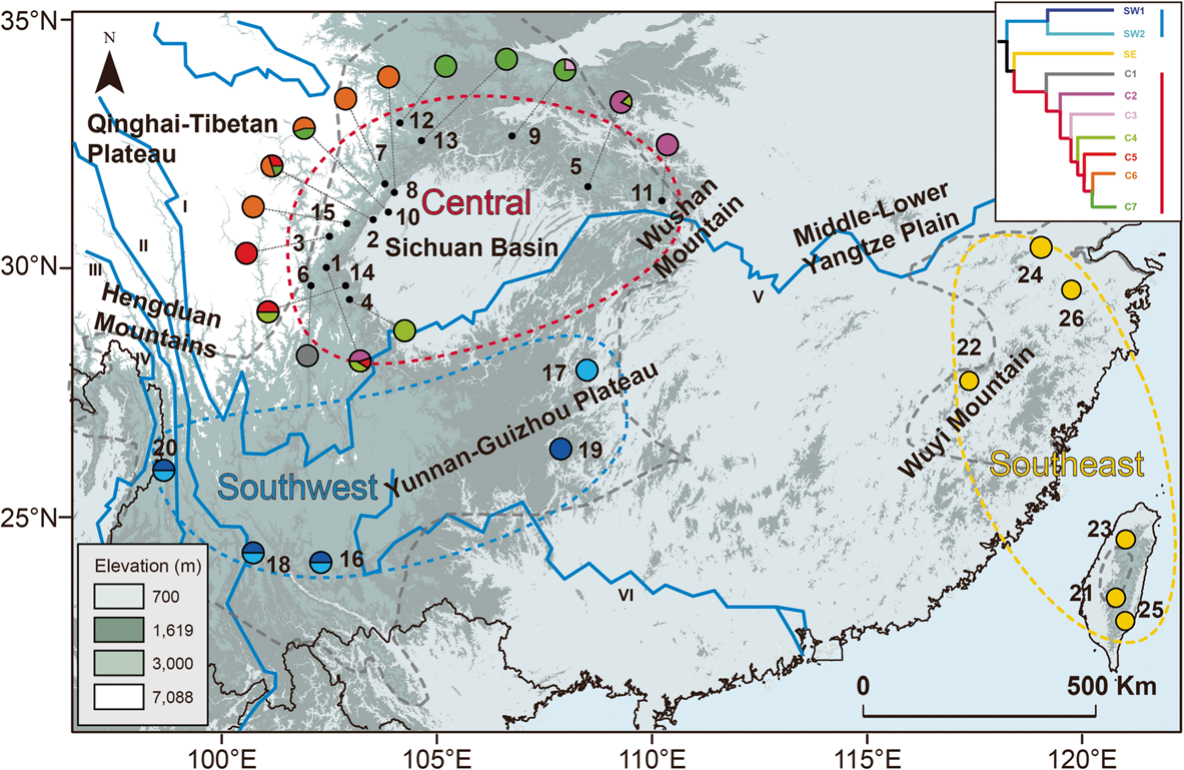
Sampling sites and extant distribution of *E. melanogaster*, with the major geographical features within the distributed area labelled. Great rivers in this area are indicated by Roman numerals: I, Jinsha River; II, Lancang River; III, Nu River; IV, Dulong River; V, Yangtze River; and VI, Pearl River. The locality codes and coordinates are presented in Additional file 1: Table S1. The elevation is shown with a legend in the bottom left. The colours of the sampling sites indicate mitochondrial clades and correspond to the colours in the phylogenetic tree shown in Fig. 2 (shown here in the top-right frame). The altitude information is freely available at http://www.worldclim.org/, and the base map from Esri Content Packages

*Eothenomys* is a typical endemic genus of the South China mountainous regions [17]. Most species within this genus are distributed within a very narrow range and represent one of the rapid radiations of the QTP and HM (also see [18]). However, *E. melanogaster* is unique in this genus; it has a much wider distribution range across the whole of South China, extending to Taiwan Island. As a montane specialist, *E. melanogaster* has a known altitudinal range from 700 to 3,000 m [19]; environments with too low or too high elevations are not suitable. Therefore, *E. melanogaster* has a prominently disjunct distribution in South China (Fig. 1), which makes it an interesting species for investigating effects of geological and climatic events on the genetic patterns of species across South China.

In this study, we investigated the population genetic structure of *E. melanogaster*, historical divergence scenarios (including patterns of diffusion and demographic variation, divergence time between clades, and divergence scenarios based on ecological niche modelling, ENM) and the effects of isolation by distance (IBD) and isolation by environment (IBE) by assessing the relationships between geographical distance/ecological vicariance and genetic distance in *E. melanogaster*. We aimed to answer the following questions: 1) What is the main cause of the disjunct distribution of *E. melanogaster*: geological events or climatic oscillations? How is the genetic structure of *E. melanogaster* shaped among and within different areas (such as the YGP and Taiwan Island)? 2) As a representative of the South China hotspot, can *E. melanogaster* inform us about concerted evolution within this community in terms of the relative roles of geological events vs. climatic oscillations? Considering the cold-adaption trait, is genetic diversity within *E. melanogaster* representative of the diversification of extant cold-adapted species through this region? Based on these two broader questions and previous studies, we hypothesized that the tectonic events since the Late Pliocene induced intraspecific divergence followed by gene admixture during the climatic oscillations.

## Methods

### Ethics statement

We carried out this study strictly following to the animal research protocol IOZ-2006 approved by the Animal Care Committee of Institute of Zoology, Chinese Academy of Sciences (IOZ, CAS). *Eothenomys melanogaster* individuals were captured with permission of the local Protection and Research Centre and the Forestry Administration of China. All of the individuals were captured using cages and then immediately euthanized by cervical dislocation. All efforts were made to minimize potential pain and suffering. Intact skulls of all the captured individuals were collected and preserved in 100% ethanol in the field and cleaned after the field survey for further taxonomy identification. Muscle tissues to be employed for molecular analysis were collected and preserved in 100% ethanol. All specimens and skulls collected in the field were deposited in the mammal collections of the National Zoological Museum of China (IOZ, CAS), Sichuan Academy of Forestry, and Kunming Natural History Museum of Zoology (Institute of Zoology, Chinese Academy of Sciences) (KIZ, CAS).

### Sampling, DNA extraction and gene sequencing

We sampled 241 individuals at 26 localities (Fig. 1 and Additional file 1: Table S1). We amplified a total of six genes: the mitochondrial cytochrome b (*cytb*) gene, the nuclear breast cancer (*BRCA1*) gene, the external transcribed spacer 2 (*ETS2*) gene, the growth hormone receptor (*GHR*) gene, intron 1 of the glucose-6-phosphate dehydrogenase (*G6pd*) gene, and exon 1 of the interphotoreceptor retinoid-binding protein (*IRBP*) gene. In addition, we used two sequences of *cytb* obtained from GenBank (accession numbers: AY426681 and AY426682 [17]). The primers used were as follows: *cytb*: H15985/L14734 [20]; *BRCA1*: F180_arv/R1240_arv [21]; *ETS2*: ETS2F/ETS2R [22]; *GHR*: GHR5 forward/GHR6 reverse [23]; *G6pd*: G6pdint1L/G6pd-int1H [24]; and *IRBP*: IRBP217/ IRBP1531 [25]. *cytb* from museum collections was amplified with primers described by Petrova et al. [26].

Polymerase chain reaction (PCR) amplifications were performed in a 25 μl volume with 0.25 μl Taq polymerase (Takara Shuzo Co. Ltd., Otsu, Japan), 3 μl of DNA template (~ 60 ng/μl), 2.5 μl 10× PCR buffer, 2 μl dNTPs (2.5 μM), and 1 μl primers (10 μM). The reactions were adjusted to a final volume of 25 μl with ddH_2_O. The PCRs were performed using the following procedure all the markers: 94 °C for 10 min; 35 cycles of 1 min at 94 °C, of 45 s at 54 °C, and of 90 s at 72 °C; followed by a final extension step at 72 °C for 10 min. The complete sequences were assembled using BIOEDIT 7.2.5 [27] and aligned using MUSCLE implemented in MEGA6 [28]. Considering that mitochondrial DNA may experience substitution saturation because of its rapid evolutionary rate, we assessed substitution saturation using the test described by Xia et al. [29] as implemented in DAMBE 6 (reference: Assessing substitution saturation with DAMBE) [30].

### Phylogenetic and divergence time analysis

To evaluate the intraspecific phylogenetic relationships among the samples, individual gene trees were reconstructed based on *cytb*, each nDNA, and concatenated *cytb* and nDNA genes using Bayesian inference (BI) methods in MrBayes 3.2.5 [31] and maximum likelihood (ML) methods in RAxML v.8.2.10 [32]. The best fit model of nucleotide substitution for each locus was selected using MrModeltest v2.3 [33]. Two parallel runs of one cold and three heated Markov chain Monte Carlo (MCMC) analyses were performed for 15 million generations or more, with trees sampled every 1,000 generations to produce convergence (SD < 0.01). The first 25% of the Markov chain samples (*N* = 20,000) were discarded as burn-in, and the remaining samples were used to generate majority rule consensus trees. ML phylogenies were inferred using a GTRGAMMAI model of evolution and 1,000 bootstrapping replicates. Final trees were then viewed in FigTree 1.4.2 (available at http://tree.bio.ed.ac.uk/software/fig-tree/). To better visualize the relationships among haplotypes, median-joining (MJ) networks [34] were generated using *cytb* in POPART 1.7 [35]. Gaps and missing sites in the sequences were excluded from the analysis. The pairwise genetic distances of *cytb* sequences were calculated with MEGA6 using the Kimura two-parameter (K2P) distance between and within each clade.

We estimated divergence time using BEAST 1.8.2 [36] to determine the relationship between genetic divergence and geological events. The analysis was performed using the *cytb* gene because of its higher degree of variation. We calibrated with the soft bounds of three fossil calibration points as follows, and the parameters were set to log-normal distributions with a 95% interval boundary. Fossil calibration point 1: The oldest fossil of *E. melanogaster* is dated at 2.03 Ma [37]. We assigned a minimum age of 2.03 Ma [offset = 1.806; log (mean) = 0.37; median = 2.030; 97.5% quantile = 3.399]. Fossil calibration point 2: The occurrence time of *Eothenomys* is dated at the Late Pliocene (3.60–2.58 Ma) [37]. We assigned a minimum age of 3 Ma [offset = 3.0; log (mean) = 0.6; median = 3.364; 97.5% quantile = 5.583]. Fossil calibration point 3: A Russian fossil of the most recent common ancestor (MRCA) of *Myodes* dated to at least 2.6 Ma [38] used by Kohli et al. [39]. We assigned a minimum age of 2.60 Ma [offset = 2.60; log (mean) = 0.20; median = 2.721; 97.5% quantile = 3.461]. The outgroups are described in Additional file 1: Table S2. Prior to divergence calibration, the *cytb* clock model was selected based on marginal likelihood estimated (MLE) from stepping stone and path sampling [40] with 100 path steps, 1 million iterations and samples every 1,000 generations and was run twice to ensure convergence. According to the MLE analysis, a relaxed log-normal clock (log marginal likelihood = − 6056.96/− 6058.59) was favoured over a strict clock (log marginal likelihood = − 6128.77/− 6131.26) for the *cytb* dataset.

To estimate the species tree and test the clade divergence revealed in the BI tree, concatenated *cytb* and nDNA sequences were analysed using *BEAST [41], which was implemented in BEAST 1.8.2. The substitution models were unlinked, and the substitution parameters were set according to the MrModeltest results. We chose Yule Process species tree priors. Nuclear gene clock models were assessed using the *ucld.stdev* parameter based on preliminary runs in which the uncorrelated relaxed log-normal clock prior was applied and the resulting distribution examined, as recommended in the program documentation [42, 43]. The uncorrelated relaxed log-normal clock was set for all loci. The MCMC chains were run for 200 million generations, with sampling every 5,000 generations. The convergence of the MCMC chains was examined in Tracer 1.6, and the first 25% of runs were discarded as burn-in. The trees and posteriors were displayed and edited in FigTree 1.4.2. To test the species delimitation, we used BP&P 3.2 [44] with all samples and all loci. Reversible-jump Markov chain Monte Carlo (rjMCMC) analyses were run for 100,000 generations with a burn-in phase of 8,000. We used a gamma prior G (2, 1,000) on the population size parameters (θs) and a gamma prior G (2, 2,000) on the age of the root in the species tree (τ_0_). The other parameters were assigned as the prior defaults [45]. Each analysis was run twice with different starting seeds to ensure consistency. In addition, the species tree analyses were performed to avoid incomplete lineage sorting if we observed congruent structure between the concatenated gene tree (mtDNA+nDNA) and the species tree.

### Demographic analyses and genetic indices visualization

The general patterns of diversity in the mtDNA and nuclear loci, we calculated the nucleotide diversity (π), the mean number of pairwise differences (k), the number of haplotypes (nh) and the haplotype diversity (h) for each clade of mtDNA and all individuals of mtDNA and nuclear loci using DnaSP 5.10.01 [46]. The significance was tested using 10,000 permutations in Arlequin 3.0 [47]. Tajima’s *D* [48] and Fu’s *Fs* [49] were conducted to test the neutrality and demographic history based on *cytb* and nuclear loci in DnaSP with 10,000 bootstrap replicates. Significant negative values for Tajima’s *D* and Fu’s *Fs* generally indicated a recent demographic expansion.

To better visualize the relationship between geographical pattern and genetic diversity/genetic divergence, the nucleotide diversity (π) and pairwise genetic distance were spatially interpolated using the Kriging method [50] implemented in the ‘Spatial Analyst Tools’ of ArcGIS 10.0 (ESRI, Redlands, CA, USA). We sampling the localities contained within a buffer of 1° radius around each sampling locality to include at least two individuals per locality in each genetic diversity calculation. We thus sampled a total of 25 localities. The results were masked with current suitable habitat estimated in MAXENT with the “10% logistic threshold” (see below).

We used BEAST to estimate molecular-based demographical fluctuation based on mtDNA only. The demographic variation was estimated using extended Bayesian skyline plots (EBSP), with three clades analysed separately. The analyses were run for 50,000,000 generations for each clade; each clade was sampled every 5,000 generations. All samples were included, and the *ucld.mean* parameter was used as previously estimated in the time calibrating analysis. The final convergence was assessed with effective sample size (ESS) in Tracer v1.6 [40]. An ESS value above 200 was considered acceptable.

### Reconstruction of historical scenarios

We implemented the discrete phylogeographical analysis in BEAST v 2.3.7 [51] to examine range expansion among the study sites through time. We used haplotype data sets with one individual per haplotype per locality to achieve better convergence and reduced the dataset to 104 individuals with *cytb*. The time calibrating also used the *ucld.mean* parameter same as for the EBSP analyses. The analysis was run for 30,000,000 generations, with sampling every 3,000 generations. Convergence of runs and thus support for the inferred ages of migration events was achieved by ensuring that the ESS for the ‘geotreelikelihood’ prior was greater than 200 in the log file. The spatial-temporal diffusion pattern was then reconstructed using Time Slicer in SPREAD v1.0.6 [52]; and GOOGLE EARTH (Google, California, USA, available at http://google.com/earth/) was used for the final visualization.

We reconstructed range variation during the Last Glacial Maximum (LGM) to estimate the climatic effect of Pleistocene ice periods. We obtained climatic data from the current conditions and the LGM (~ 21,000 years before present). We used two models of the LGM climate: the community climate system model [CCSM ver. 3; [53]] and the model for interdisciplinary research on climate [MIROC ver. 3.2; [54]]. Nineteen bioclimatic variables at a resolution of 2.5 arc-min for each period were downloaded from the WorldClim database version 1.4 (available at http://www.worldclim.org/) [55]. To reduce the amount of computation, we masked all variables to include only 60° to 125°E and 15° to 35°N. All species occurrence data were collected from our sampling localities, museum records from the National Zoological Museum of China, and occurrence records from the Global Biodiversity Information Facility database (available at http://www.gbif.org/). Finally, 139 localities were used in analysis. As the influence of the effect of over-fitting, we firstly tested the correlation among climatic variables and removed one variables if two variables were highly correlated (Pearson’s correlation > 0.8). We further performed model fitness analysis in ENMTools 1.4.4 [56] by examining beta regulation values from 1 to 20. We finally kept the climatic variables of BIO1, BIO2, BIO4, BIO12, BIO14, BIO15, BIO18 and BIO19, with a beta value of two. Then, we constructed the model by randomly selecting 80% of the occurrence data, and 20% of the data were left to test the accuracy of the model in MAXENT v3.3.3 k [57]. The setting of parameters used the default convergence threshold (10^− 5^), 2,000 maximum iterations and 10 replications. MAXENT was performed to test the model by calculating the average area over ten replications under the receiver operating characteristic curve (AUC) and the binominal probabilities indicating the predictive ability of the model. We calculated the average elevation of suitable habitat during the LGM and the current period in ArcGIS 10.0. The area of suitable habitat was based on a 10% logistic threshold from the MAXENT result.

### Examination of the effects of IBD and IBE

The effective population size (Θ = effective population size, N_e_ x mutation rate, μ) and effective migration rates (M = migration rate, m/mutation rate, μ) between sample sites were calculated using MIGRATE 3.6.11 [58, 59] to estimate the gene flow among the three maternal clades that were found. To eliminate bias caused by unequal population size, we subsampled the dataset to include 45 individuals, with 15 individuals per clade estimated based on all loci. MIGRATE used BI with long chains (500,000 steps sampled, 5,000 steps recorded) and 1,000 burn-in per chain. The mutation scaled M entering and leaving each population per generation and the mutation scaled Θ were estimated applying the Bayesian search strategy to determine whether there was asymmetrical gene flow between populations. N_e_m was calculated by multiplying M and Θ.

We implemented two analyses to assess the relationships between geographical distance/ecological vicariance and genetic distance. We first examined the effect of IBD, which was estimated by the correlation between the genetic distance and geographic distance (Euclidean distance) of each pair of localities. A Mantel test was applied to calculate correlations using the package VEGAN [60] in R [61]. Second, we tested ecological vicariance (IBE) between clades using spatial evolutionary and ecological vicariance analysis (SEEVA; [62]). We evaluated four ecological characters: mean annual temperature (BIO1), annual precipitation (BIO12), temperature seasonality (standard deviation*100, BIO4), and precipitation seasonality (coefficient of variation, BIO15). Climatic traits of each locality were extracted using ArcGIS 10.0. SEEVA was then used to divide these quantitative traits into four quartiles and calculate the correlations between ecological shifts and phylogenetic splits represented by Fisher’s exact tests and divergence indices (D).

## Results

### Phylogenetic and divergence time analysis

A total of 241 individuals from 26 localities were sampled, including data from GenBank (Fig. 1). We obtained six genetic markers from PCR: 1,074 base pairs (bp) of the *cytb* gene, 930 bp of the *BRCA1* gene, 935 bp of the *ETS2* gene, 777 bp of the *GHR* gene, 633 bp of intron 1 of the *G6pd* gene, and 1,210 bp of exon 1 of the *IRBP* gene. The sequence dataset generated herein is available in GenBank, and the accession numbers are listed in Additional file 1: Table S1. The saturation test showed that none of the genes we used indicated significant saturation (I_ss_ < I_ssc_, *P* < 0.001, data not shown).

**Fig. 2:**
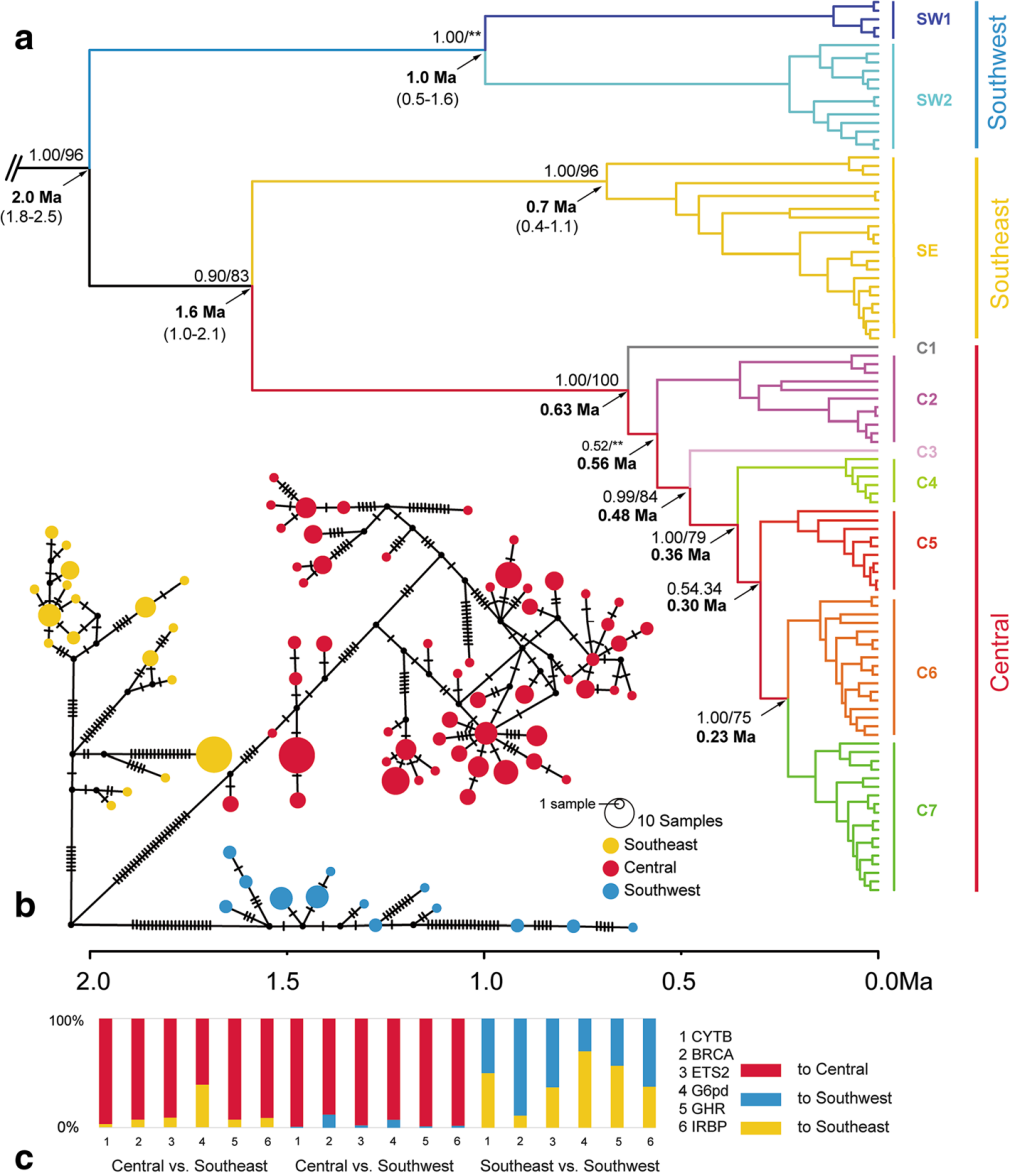
**a** Phylogenetic tree and divergence time estimation based on BEAST analysis of the *cytb* haplotypes of *E. melanogaster*. The numbers beside the nodes are posterior probabilities and bootstrap, and those below the nodes are divergence times estimated by BEAST (Ma, 95% HPD in brackets). **b** Median-joining network based on *cytb* with node sizes proportional to the frequencies of haplotypes*.* The number of hatch marks between haplotypes is proportional to the number of mutational changes. Dots represent undetected haplotypes. **c** Gene flow between maternal clades as estimated by MIGRATE (represented in %)

BI and the ML trees based on *cytb* only, concatenated *cytb* and nDNA clearly indicated three clades: Southwest (SW), Southeast (SE) and Central (C) clade (Fig. 2a). The SE clade and C clade showed a closer relationship. The haplotype network showed the same result (Fig. 2b). In the C clade, the distribution of subclades was similar to a ring pattern around the Sichuan Basin, with early-diverged subclades at both ends (subclades C1, C2, C3, and C4) and a recent diversification in the northwest margin of the basin (subclades C5, C6, and C7; Fig. 1). However, the phylogenetic relationships based on different nuclear loci were reconstructed with low support except for that those based on *ETS2* (see Additional file 2: Figure S1 for details). K2P analysis showed that the *cytb* genetic distance between different clades ranged from 6.44% to 8.04%.

The divergence time data indicated that a hierarchical pattern had occurred since the most recent ancestor of the entire in-group, which was estimated to have existed at 2.0 Ma [1.8–2.5 Ma, 95% highest posterior density (HPD); Fig. 2a], placing the ancestry of all living representatives of the species in the Early Pleistocene. The SW clade was the first to diverge, and the subsequent divergence events of the SE and C clades occurred at 1.6 Ma (1.0–2.1 Ma, 95% HPD).

In the BP&P species delimitation analysis, three clades were strongly supported (posterior = 1.0). The *BEAST and BP&P tree showed inconsistent structure with the BI and ML tree for concatenated *cytb* and nDNA (Additional file 3: Figure S2). Based on the *BEAST and BP&P tree, the SW and C clades showed a closer relationship to each other than to the SE clade, and the SE clade was the first to diverge. However, the posterior probabilities were low for this structure in these two analyses.

### Demographic analyses and genetic indices visualization

**Fig. 3:**
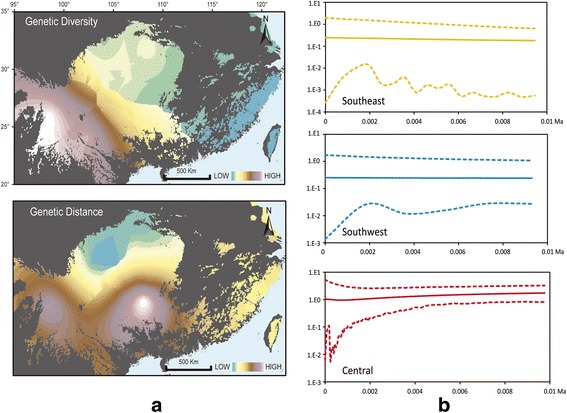
**a** Interpolated distribution pattern of genetic diversity and genetic divergence. The distribution range was masked with habitat estimated by MAXENT with a range of 10% logistic threshold. **b** Extended Bayesian Skyline plots for all the main clades of *E. melanogaster*. The solid lines indicate the median value of effective population size; the dashed lines denote the 95% highest posterior probability interval. The base map comes from Esri Content Packages

The SE clade had the highest mitochondrial nucleotide diversity (2.47%), the C clade had the second highest (1.79%), and the SW clade had the lowest (1.54%) (Table 1). The genetic diversity of nuclear loci ranged from 0.6% to 0.23%. The interpolated genetic diversity showed an upward trend from east (0.062%) to west (6.52%), whereas genetic distance showed deeper divergence from north to south (Fig. 3a). The genetic distance plot illustrates the degree of divergence between each clade. The values of Tajima’s *D* and Fu’s *Fs* were mostly negative but non-significant, with the only exception being the *D* value (significantly negative) of *IRBP* and the *Fs* values (significantly negative) of *BRCA1*, *ETS2*, and *IRBP* (Table 1). The demographic scenario obtained by the EBSP showed that the three clades have generally remained stable since 0.01 Ma, although the C clade showed a slight decline (Fig. 3b).

**Table 1:** Genetic diversity and neutrality test estimates based on mtDNA and nDNA for all individuals and each clade

Marker	Clade	Number	nh	s	Pi (%)	h	Pairwise difference (k)	Tajima’s *D*	Fu’s *Fs*
*cytb*	all	241	86	91	4.32	0.98	31.38	−0.13	−1.43
	SE	49	20	128	2.47	0.88	25.52	−0.11	−0.44
	C	163	61	54	1.79	0.98	16.30	−0.09	−0.57
	SW	29	14	–	1.54	0.91	12.16	−0.11	− 0.04
*BRCA1*		62	19	19	0.23	0.86	2.02	−0.08	**− 0.08**
*ETS2*		71	24	32	0.60	0.87	4.86	−0.08	**− 0.36**
*G6pd*		48	8	7	0.32	0.81	1.51	−0.09	0.27
*GHR*		68	16	21	0.59	0.87	4.37	−0.10	−0.16
*IRBP*		65	32	39	0.40	0.96	3.51	**−0.07**	**−0.31**

The main clades, numbers of individuals (n), number of haplotypes (nh), number of polymorphic (segregating) sites (s), nucleotide diversity (Pi), haplotype diversity (h), and mean number of pairwise nucleotide differences (k). Values in bold indicate significance based on the *P* value

### Reconstruction of historical scenarios

**Fig. 4:**
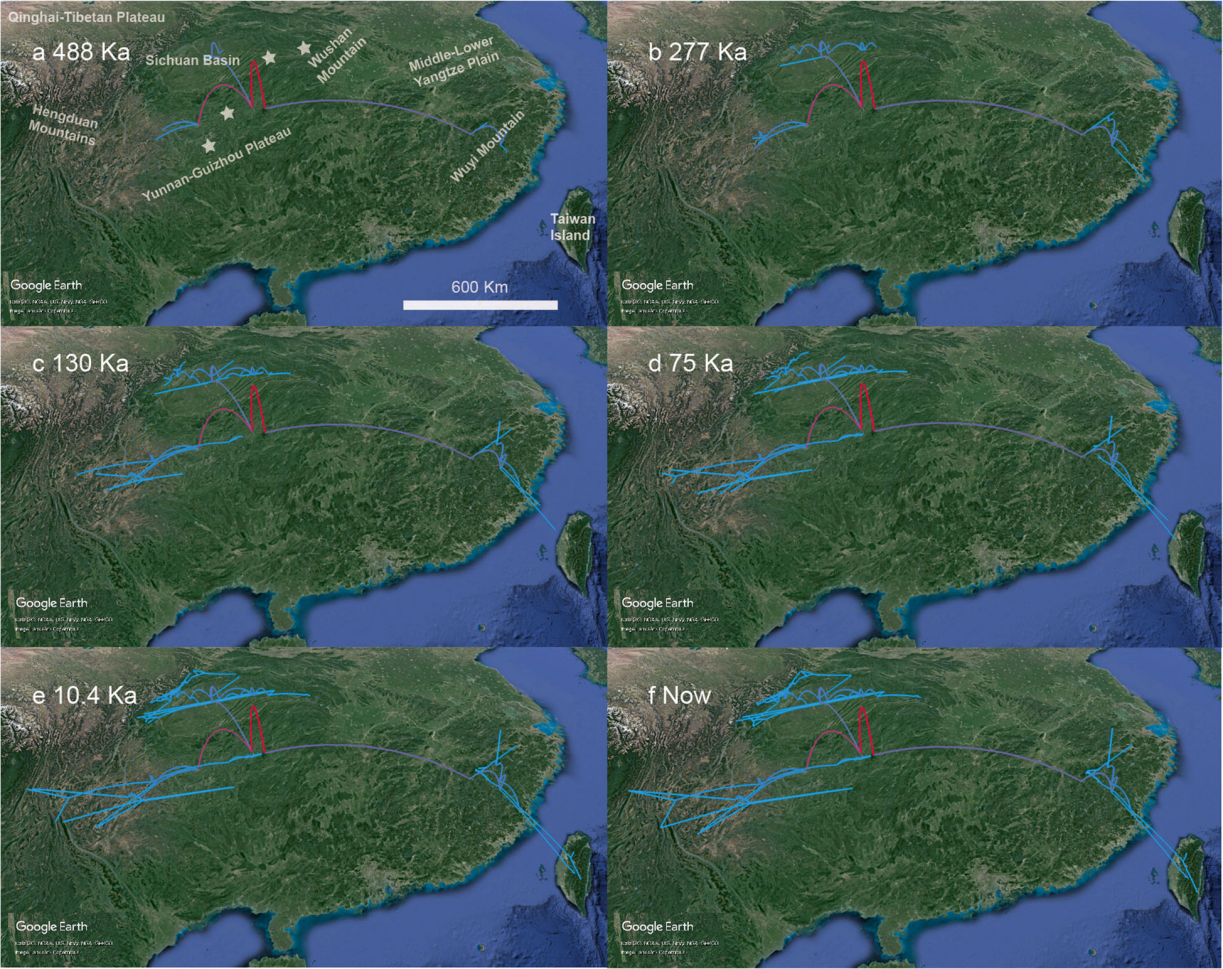
Results of the phylogeographic diffusion analysis of time points at (**a**) 488 Ka when *E. melanogaster* originally covered its extant distribution range, (**b**) the beginning of the penultimate glaciation period (277 Ka), (**c**) the end of the penultimate glaciation period (130 Ka, (**d**) the beginning of the last glaciation period (75 Ka), (**e**) the end of the last glaciation period (10.4 Ka), and (**f**) present. Asterisks represent the fossil records of *E. melanogaster*. The red line represents the ancient time and the blue line represents the current time. The map used here is from Google Earth

The phylogeographic diffusion analysis reconstructed a pattern suggesting that *E. melanogaster* originated near the YGP (Fig. 4) and that the three clades (SW, C, and SE) then likely derived from YGP, Sichuan Basin and Wuyi Mountains, respectively. The earliest fossil record was found in the Wushan Mountains, near YGP. Many locations experienced multiple colonization events and all three clades spread rapidly in the meantime. *E. melanogaster* was inferred to have migrated twice to Taiwan Island during the glaciation periods. The ancestors of the twice colonization have appeared at the end of penultimate glaciation period (130 Ka) (Fig. 4c) and the colonization events have finished at the end of last glaciation period (10.4 Ka) (Fig. 4e) [63].

**Fig. 5:**
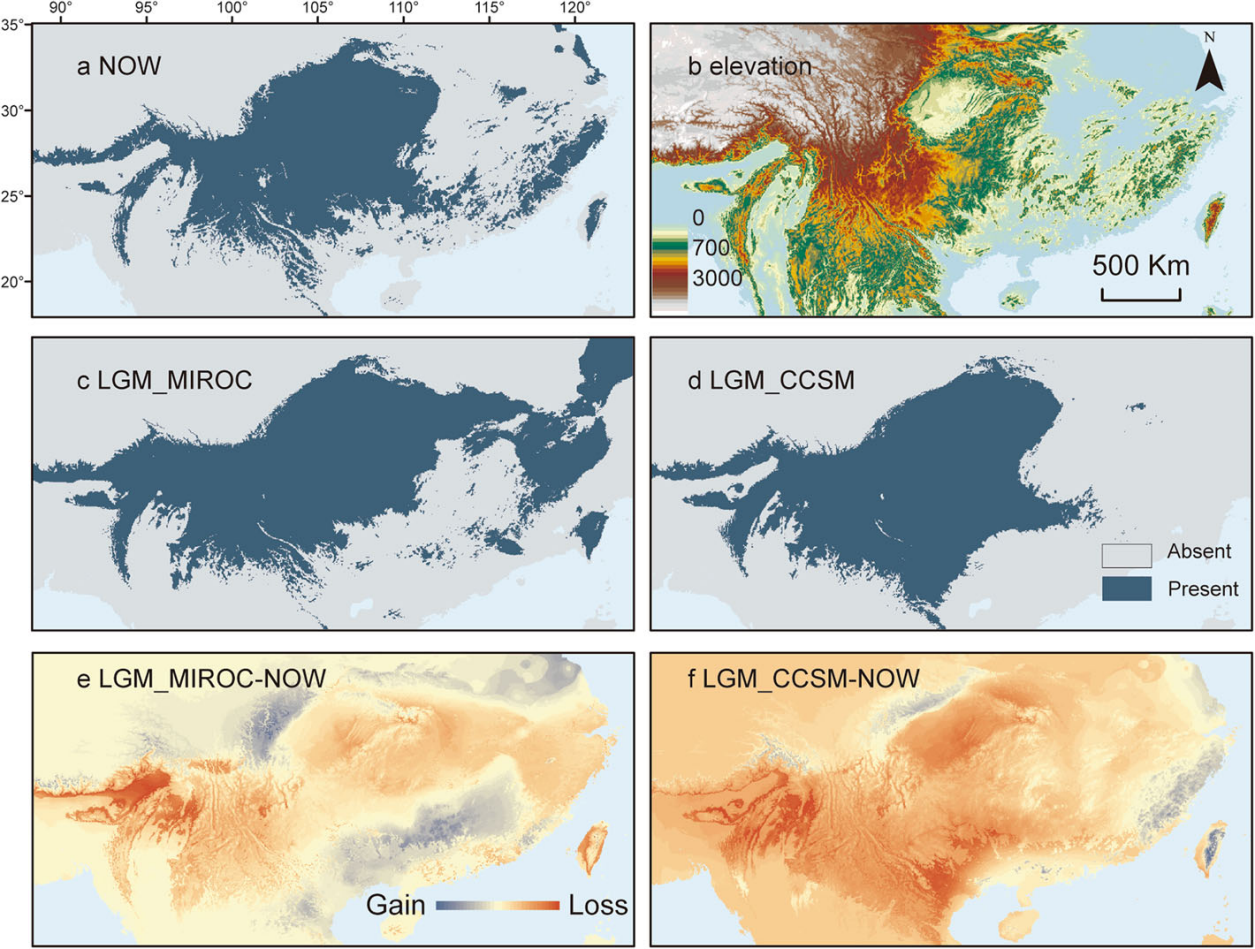
Ecological niche modelling of suitable habitats for *E. melanogaster* generated by MAXENT. The simulations were conducted based on the climatic conditions of (**a**) the current climate, (**c**) the Last Glacial Maximum (LGM) of the MIROC model and (**d**) the LGM of the CCSM model. **b** is the elevation distribution of this area. The continental margin in the LGM differed from the others because of the lower sea level at the time. The suitable habitat was based on the 10% logistic training threshold estimated by MAXENT. Differences in occurrence between the two periods are shown in (**e**) and (**f**). Gains (blue) mean the increase of suitable habitat in current condition, and losses (red) mean opposite. The altitude and bio-climate information are freely available at http://www.worldclim.org/, and the base map from Esri Content Packages

In the current ENM, the area under the ROC (Receiver operating characteristic) curve (AUC; Area under curve) was close to one (0.997 ± 0.002), indicating better than random prediction (0.5 = random, 1 = maximum), and the binomial probability was *P* < 0.001. The ENM shows the importance of suitable habitats and elevations (Fig. 5). The suitable habitats showed more fragmentation in current conditions, especially in low-latitude regions. *E. melanogaster* currently tends to live at higher elevations (mean elevation calculated based on 10% logistic threshold: now: 1,397.033 m), whereas its suitable habitat was lower in the north and expanded southward during the LGM (MIROC 1,134.238 m, CCSM: 1,236.908 m).

### Examination of the effects of IBD and IBE

Gene flow among the three clades showed similar patterns for all six genes: the SW and SE clades produced migrants entering the C clade, and the reverse gene flows from the C clade to the other two clades were much lower (Fig. 2c and Additional file 1: Table S3). Gene flow between the SW and SE clades was bidirectional. A Mantel test showed strong correlations between geographical distance and genetic distance (*r* = 0.4363, *P* = 0.0001).

**Fig. 6:**
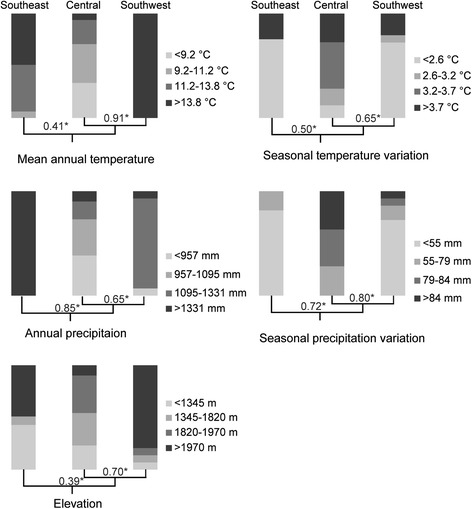
The results of the spatial evolutionary and ecological vicariance analysis (SEEVA) of *E. melanogaster* using mean annual temperature, annual precipitation, seasonal climatic variation and elevation coded as four quantitative section states. The total height of each histogram bar equals 100% of the observations for each clade, and the greyscale of the histograms represents the four different states. The underlined phylogeny was based on the Bayesian mitochondrial phylogeny and the species tree estimated by BP&P and *BEAST. The nodal values represent the Index of divergence (D), and asterisks indicate significant differences between sister groups using a Bonferroni criterion of *P* ≤ 0.05

Based on SEEVA the three clades all showed significant divergence in all the examined ecological variables (Fig. 6). The C clade was generally found at a lower level of mean annual temperature with higher seasonal variation and a lower level of precipitation with higher seasonal variation, whereas the SW and SE clades lived in habitats with a higher mean annual temperature and precipitation with less seasonal variation. Southern populations were located at higher elevations than northern populations (Fig. 6), a pattern that is possibly related to the latitudinal gradient of the distribution of each clade.

## Discussion

### Intraspecific diversification with disjunct distribution

Our study performed comprehensive phylogeographic analyses to infer the evolutionary history of *E. melanogaster* throughout the Quaternary and to obtain insights into the processes that shaped its disjunct distribution pattern. The mtDNA analyses revealed a clear hierarchical pattern of divergence with geographical structure for *E. melanogaster* (Fig. 2a). Three reciprocally monophyletic maternal clades were supported: SE clade (consisting of individuals from Taiwan Island and southeast coastal China), C clade (individuals from mountainous areas surrounding the Sichuan Basin and the southeast edge of the QTP) and SW clade (individuals from the YGP and the southern part of the HM) (Fig. 1). The analyses, which included analyses of nuclear loci, all identified these three genetic clades but showed incongruence between the *cytb* and nDNA trees. This discordance in inference between mtDNA and nDNA genes is likely attributed to recent mitochondrial admixture or sex-biased gene flow in this species [64, 65]. However, *E. melanogaster* shows no significant recorded difference in dispersal capability between males and females [66]. Our nDNA tree indicated a geographical structure similar to that obtained based on mtDNA, and the support values of the three clades were fairly high, arguing against sex-biased gene flow [67]. Furthermore, the detected bidirectional gene flow between the SW and SE clades also argues against sex-biased gene flow. The only difference in structure between the mtDNA and nDNA phylogenies was in the position of the SE and SW clades and secondary mitochondrial admixture is the most likely reason (Fig. 2 and Additional file 2: Figure S1). Furthermore, incomplete lineage sorting was not observed in our study, as congruent structure was observed between the concatenated gene tree (mtDNA + nDNA) and species tree (BP&P and *BEAST; Additional file 2: Figure S1 and Additional file 3: Figure S2).

The phylogeographic diffusion analysis reconstructed a pattern showing that *E. melanogaster* originated from around the YGP (Fig. 4a), which is consistent with the earliest fossil record (2.03 Ma) of *E. melanogaster* [37]. The first divergence between the SW clade and the other two clades occurred during the Early Pleistocene (2.0 Ma). The SW clade could communicate with the C clade until the late Early Pleistocene, when the rapid uplift of the QTP and the YGP facilitated the development of the palaeo-Jinsha River that drained into a palaeolake in the Sichuan Basin [68]. During the boundary of the Early Pleistocene and Middle Pleistocene (0.7 Ma at the latest), the Wushan Mountains were intersected, which resulted in the formation of the modern Yangtze River [1]. This time period coincided with the Kunlun-Huanghe Movements, when the QTP and adjacent mountainous regions were strongly uplifted [69]. The connection of the palaeo-Yangtze River contributed to the split and further prevented gene flow between the SW and C clades. The SE and C clades subsequently diverged at approximately 1.6 Ma, when the elevation of the QTP during that time was low (< 2,000 m) [70, 71] and the plains in the southeast were at a higher elevation (the Middle-Lower Yangtze Plain had subsided approximately 20–360 m during the Quaternary) [1]. Moreover, a Mantel test showed a strong correlation between geographical distance and genetic distance. Therefore, the divergence of the SE clade was more likely a consequence of long-distance dispersal than of the vicariance induced by the landscape. The SE clade resided in the Wuyi Mountains, flourished and then spread to adjacent areas as the mountains in the southeast continuously rose.

The results of the phylogeographic diffusion analysis indicated that the distribution of *E. melanogaster* had already covered its extant distribution range by approximately 488 Ka (Fig. 4a). Along with the continuous uplift of the mountains and sedimentation of the plains and basins of South China, *E. melanogaster* gradually adapted to medium-high elevations and a cold environment, maintained and centred on the YGP, the mountainous areas surrounding the Sichuan Basin and the Wuyi Mountains (Fig. 1). Populations from lower elevations might have become extinct or subdivided because of unsuitable environments [72]. Ecological divergence further reinforced the genetic differentiation that resulted from the geographical isolation [43]. The IBE results indicated distinct vicariance of ecological traits among these three clades, especially between the C clade and each of the other two (Fig. 6). The C clade showed wider ecological niche utilization. In contrast to the patterns of species that live at lower elevations, such as the grey-cheeked fulvetta and the red-headed tree babbler [9, 73], the diversification of the high-altitude cold-adapted community represented by *E. melanogaster* is deeply influenced by the Pleistocene interglacial within warm-phase refugia, followed by secondary contact during cold phases [4, 74–76]. This pattern is also counter to the general dogma of glacial refugia and secondary contact during warm phases [77, 78]. The extant disjunct distribution pattern of *E. melanogaster* formed in response to the combination of these conditions and adds an important example to the growing literature for a full understanding of Pleistocene dynamics as a seesaw mechanism of diversification.

### Genetic admixture during Pleistocene climatic oscillations

Although the main divergence occurred as a result of IBD and geological events, Pleistocene climatic changes were profoundly imprinted in the genetic structure, as was clearly supported by the ENM results (Fig. 5). Unlike some other thermophilic species, such as four species of Passeriformes birds [2], *Quasipaa boulengeri* [12], and *Mus musculus* [79], *E. melanogaster* exhibited traits (either psychrophilic or environment dependent) that led to expansion in the glacial period and contraction in the interglacial period. This result is similar to results found for some previously studied species in this region [3]. The middle elevational environment remained suitable in each period, whereas the suitability of low and high areas fluctuated (Fig. 5e and f, respectively).

During the ice ages, populations of *E. melanogaster* shifted to lower elevations because of the expansion of habitat in low-altitude areas such as the Sichuan Basin (elevation range from 200 to 750 m) and Middle-Lower Yangtze Plain and degradation of habitat in high-altitude areas such as large mountains in the northwest of Sichuan Basin, Wuyi Mountains and the Central Mountains of Taiwan (elevations over 3,000 m). The mean elevation shifts of *E. melanogaster*-applicable habitat between the LGM and current conditions intuitively reflected the tendency that facilitated genetic exchange. In warm periods, high-altitude localities acted as “sky islands” for *E. melanogaster* and resulted in genetic divergence [7]. The high levels of subclade diversity of the C clade (Fig. 2a) can be explained by this scenario. The genetic divergence that occurred during the interglacial periods and the secondary contact that occurred during the glacial periods shaped the current genetic structure. Although the analysis of nuclear data did not yield well-resolved relationships within the C clade, the weak consistency of the nuclear data with mitochondrial subdivision implied gene flow and genetic admixture.

Populations from different sites in the SE clade showed a paraphyletic structure. According to the results of the phylogeographic diffusion analysis, the whole SE clade population originated and expanded from the Wuyi Mountains area (Fig. 4). Taiwan Island is situated 230 km from the Chinese mainland. During the Pleistocene glaciations, the sea level of the Taiwan Strait fell sufficiently to create a land bridge between Taiwan and the mainland many times, which promoted contact between the organisms on both sides [1]. Populations from Taiwan Island were not isolated and repeatedly experienced colonization via the land bridge during cold periods. Similar cases of gene flow between populations in Taiwan and the mainland have been reported for two bird species [9].

Two long-distant branches with weak geographic association are found within the SW clade (Fig. 1). This pattern is associated with two distinct colonization events of two different ancestors, as indicated by the results of the phylogeographic diffusion analysis (Fig. 4). This particular genetic structure was possible due to the stable environment of the mountains of Southwest China during the Pleistocene. The phylogenetic relationships and divergence pattern revealed that the SW clade has the greatest extent of ancestral polymorphisms [77], which was also generated by the asymmetric gene flow among the three clades (Fig. 2c) [78]. Moreover, because *E. melanogaster* lives between 700 m and 3,000 m above sea level, we suspect that large mountain ridges (over 3,000 m) can act not only as corridors for this species but also as barriers. Therefore, a stable environment led to low diversification, and the dual effects of mountains shaped distant phylogenetic relationships within clades.

### Demographic history and diversity patterns

Haplotype diversity (h) and nucleotide diversity (Pi) can reveal the general demographic history of populations [80]. The C clade showed high h and low Pi values, which suggests rapid population growth from an ancestral population (Table 1). Moreover, a similar star-shaped network and small recent fluctuations suggests that the C clade might have experienced recent expansion [14]. In contrast, the SE clade has low h and high Pi values due to a short bottleneck in a large ancestral population [81]. However, the EBSP shows that the SE clade was stable during the last 0.1 Ma (Fig. 3b). In addition, low h and high Pi implies possible admixture of samples from small, geographically subdivided populations [82]. This finding is congruent with the repeated colonization and weak phylogeographic pattern. However, both the SE and C clades show low genetic diversity within localities, which likely indicate that the isolation effect of low elevations led to greater genetic exchange within localities rather than among them [83].

Among the three clades, the SW clade shows the lowest overall genetic diversity and genetic divergence (Table 1); however, the genetic diversity and genetic divergence of each locality within this clade are the highest (Fig. 3a). The complex terrain of Southwest China and stable historical environment facilitated stable species evolution without severe fluctuations [3, 9] and preserved the high levels of genetic diversity. The EBSP analysis indicate that the SW clade has remained stable over the last 0.1 Ma (Fig. 3b). However, further sample collections in the area of the SW clade are needed to facilitate the understanding of the intra-clade phylogeographic pattern and demographic history.

## Conclusions

In this study, comprehensive phylogeographic analyses were performed to infer the evolutionary history of *E. melanogaster* throughout the Quaternary and to obtain insights into the processes that shaped this species’ disjunct distribution pattern. The genetic analysis revealed three deeply divergent clades, Southeast, Southwest and Central, which derived from the Wuyi Mountains, the YGP and the mountains around the Sichuan Basin, respectively—mostly since the Pleistocene. IBD played important roles in early divergence, and geological events (the sedimentation of plains and the joining of palaeo-rivers) and IBE further reinforced genetic differentiation. The primary cause of the disjunct distribution in *E. melanogaster* was the preference for middle-high-altitude habitats in the current period. High-altitude localities acted as “sky islands” for *E. melanogaster* and blocked genetic exchange among populations. Pleistocene climatic cycles had various impacts on the diversification of different clades. Genetic admixture in cold periods and genetic diversification in warm periods were facilitated in inland clades but led to multiple colonization events between the mainland and Taiwan and erased genetic differentiation during cold periods.

## Additional files

Additional file 1: **Table S1.** Sample information and the GenBank accession numbers. Sampling information and the GenBank accession numbers of all sequences of *Eothenomys melanogaster* used in the study. **Table S2.** Outgroups of phylogenetic analysis. Outgroups used for fossil calibration and phylogenetic analyses of *E. melanogaster*. **Table S3.** Gene flow of three maternal clades. Gene flow of three maternal clades of *E. melanogaster* estimated by MIGRATE. C represents the Central Clade, SE represents the Southeast Clade and SW represents the Southwest Clade. (DOCX 32 kb)

Additional file 2: **Figure S1.** Bayesian phylogenetic trees of nuclear loci and species tree. Bayesian phylogenetic trees of nuclear loci and species tree estimated by BP&P and *BEAST for *E. melanogaster*. Red indicates individuals belonging to the Central clade; blue indicates individuals belonging to the Southwest clade; and yellow indicates individuals belonging to the Southeast clade. Values beside branches indicate Bayesian posterior probability. (TIFF 24617 kb)

Additional file 3: **Figure S2.** Phylogenetic trees of concatenated cytb and nDNA genes. Phylogenetic trees of concatenated *cytb* and nDNA genes for *E. melanogaster*. Values beside branches indicate posterior probability and bootstrap. (TIFF 28922 kb)

## Abbreviations

BI: Bayesian inference
C: Central
HM: Hengduan Mountains
IBD: Isolation by distance
IBE: Isolation by environment
IOZCAS: Institute of Zoology, Chinese Academy of Sciences
ML: maximum likelihood
MLE: Marginal likelihood estimated
QTP: Qinghai-Tibetan Plateau
SE: Southeast
SW: Southwest
YGP: Yunnan-Guizhou Plateau
