# Supplementary material for: Disjunct distribution and distinct intraspecific diversification of Eothenomys melanogaster in South China
Source: BMC Evol Biol. 2018 Apr 10;18:50. doi: 10.1186/s12862-018-1168-3 (PMC5894153; doi:10.1186/s12862-018-1168-3)
Supplement: Supplementary file 1 — Table S1. Sample information and the GenBank accession numbers. Sampling information and the GenBank accession numbers of all sequences of Eothenomys melanogaster used in the study. Table S2. Outgroups of phylogenetic analysis. Outgroups used for fossil calibration and phylogenetic analyses of E. melanogaster. Table S3. Gene flow of three maternal clades. Gene flow of three maternal clades of E. melanogaster estimated by MIGRATE. C represents the Central Clade, SE represents the Southeast Clade and SW represents the Southwest Clade. (DOCX 32 kb) [file 12862_2018_1168_MOESM1_ESM.docx]

**Additional file 1**

**Disjunct distribution and distinct intraspecific diversification of *Eothenomys melanogaster* in South China**

Xue Lv, Jilong Cheng, Yang Meng, Yongbin Chang, Lin Xia, Zhixin Wen, Deyan Ge, Shaoying Liu, Qisen Yang

**Table S1** Sampling information and the GenBank accession numbers of all sequences of *Eothenomys melanogaster* used in the study.

| Code | Locality No. | Latitude | Longitude | Sampling locality | n | *Accession number* | | | | | |
| --- | --- | --- | --- | --- | --- | --- | --- | --- | --- | --- | --- |
|  |  |  |  |  |  | *CYTB* | *BRCA* | *ETS2* | *G6pd* | *GHR* | *IRBP* |
| BAX | 1 | 29.88 | 102.35 | Baoxing | 2 | KX095651-KX095652 |  |  |  |  |  |
| DJY | 2 | 31.03 | 103.63 | Dujiangyan | 10 | KX095658-KX095667 | KX095588-KX095592 | KX095402-KX095406 | KX095471 | KX095551-KX095555 | KX113380-KX113384 |
| JJS | 3 | 30.62 | 102.85 | Jiajinshan | 27 | KX095653-KX095657, KX095717-KX095737 | KX095587,KX095610 | KX095401, KX095426 |  | KX095572, KX095520, KX095521 | KX113377-KX113379, KX113407-KX113408 |
| JKH | 4 | 29.37 | 103.03 | Jinkouhe | 15 | KX095738-KX095752 | KX095616 | KX095434 | KX095495 | KX095519 | KX113414-KX113416 |
| KAX | 5 | 31.64 | 108.75 | Kaixian | 16 | KX095753-KX095767 | KX095617-KX095620 | KX095435-KX095438 | KX095496-KX095499 | KX095568-KX095571 | KX113417-KX113422 |
| LUD | 6 | 29.64 | 102.13 | Luding | 1 | KX095770 |  |  |  |  |  |
| MAX | 7 | 31.70 | 103.91 | Maoxian | 9 | KX095773-KX095780 |  |  |  |  | KX113423-KX113424 |
| MIZ | 8 | 31.52 | 104.13 | Mianzhu | 8 | KX095781-KX095787 |  | KX095440 |  |  | KX113425-KX113426 |
| NAJ | 9 | 32.67 | 106.96 | Nanjiang | 4 | KX095788-KX095791 |  |  |  |  | KX113427-KX113429 |
| PEZ | 10 | 31.23 | 103.86 | Pengzhou | 24 | KX095792-KX095811 |  | KX095441 |  |  | KX113430-KX113433 |
| SNJ | 11 | 31.47 | 110.39 | Shennongjia | 6 | KX095812-KX095815, KX113461-KX113462 | KX095621-KX095626 | KX095442-KX095447 | KX095500-KX095505 | KX095530-KX095535 | KX113434-KX113439 |
| TJH | 12 | 32.93 | 104.29 | Tangjiahe | 20 | KX095816-KX095828, KX095834-KX095840 | KX095627-KX095630 | KX095448-KX095452 |  | KX095546-KX095550 | KX113440-KX113444 |
| TJH | 13 | 32.57 | 104.80 | Tangjiahe | 5 | KX095829-KX095833 |  |  |  |  |  |
| WWS | 14 | 29.64 | 102.95 | Wawushan | 2 | AY426681-AY426682 |  |  |  |  |  |
| WOL | 15 | 30.90 | 102.98 | Wolong | 14 | KX095844-KX095857 | KX095634-KX095639 | KX095456-KX095461 |  | KX095580-KX095584 | KX113448-KX113453 |
| ESH | 16 | 24.17 | 102.41 | Eshan | 2 | KX095668-KX095669 |  |  |  |  |  |
| FJS | 17 | 27.94 | 108.61 | Fanjingshan | 21 | KX095670-KX095690 | KX095593-KX095605 | KX095407-KX095421 | KX095472-KX095486 | KX095522-KX095529, KX095573-KX095579 | KX113385-KX113399 |
| JID | 18 | 24.39 | 100.79 | Jingdong | 2 | KX095700-KX095701 |  | KX095432-KX095433 |  |  |  |
| LES | 19 | 26.38 | 108.08 | Leishan | 2 | KX095768-KX095769 |  |  |  |  |  |
| LUS | 20 | 26.01 | 98.61 | Lushui | 2 | KX095771-KX095772 |  | KX095439 |  |  |  |
| ALS | 21 | 23.51 | 120.80 | Alishan | 6 | KX095648-KX095650, KX095842-KX095843 | KX095586,KX095631-KX095632 | KX095399-KX095400, KX095453-KX095454 | KX095470,KX095508 | KX095539-KX095541, KX095542-KX095543 | KX113375-KX113376, KX113445-KX113446 |
| FUJ | 22 | 27.74 | 117.64 | Wuyishan | 21 | KX095691-KX095696, KX095702-KX095716 | KX095606,KX095611-KX095615 | KX095422,KX095427-KX095431 | KX095487,KX095490-KX095494 | KX095562,KX095563-KX095567 | KX113400-KX113403, KX113409-KX113413 |
| GUW | 23 | 24.51 | 121.11 | Guanwu | 3 | KX095697-KX095699 | KX095607-KX095609 | KX095423-KX095425 | KX095488-KX095489 | KX095518,KX095544-KX095545 | KX113404-KX113406 |
| TMS | 24 | 30.35 | 119.43 | Tianmushan | 1 | KX095841 | KX095633 | KX095455 | KX095509 | KX095561 | KX113447 |
| WUL | 25 | 22.97 | 121.11 | Wuling | 3 | KX095858-KX095860 | KX095640-KX095642 | KX095462-KX095464 | KX095510-KX095512 | KX095536-KX095538 | KX113454-KX113455 |
| YIW | 26 | 29.49 | 120.16 | Yiwu | 15 | KX095861-KX095875 | KX095643-KX095647 | KX095465-KX095469 | KX095513-KX095517 | KX095556-KX095560 | KX113456-KX113460 |

**Table S2** Outgroups used for fossil calibration and phylogenetic analyses of *E. melanogaster*.

| Species | Genbank accession number | | | | | |
| --- | --- | --- | --- | --- | --- | --- |
|  | CYTB | BRCA1 | ETS2 | G6pd | GHR | IRBP |
| *Eothenomys chinensis* | HM165433 |  |  |  | GQ374497 |  |
| *Eothenomys custos* | AY426677 |  |  |  |  |  |
| *Eothenomys olitor* | AY426690 |  |  |  |  |  |
| *Eothenomys proditor* | AY426691 |  | KJ556818 | KJ556605 |  | KJ556772 |
| *Eothenomys wardi* | JQ818224 |  |  |  |  |  |
| *Microtus agrestis* | AY167149 | KX455596 |  |  | KX455568 | JX457685 |
| *Microtus californicus* | AF163891 | KX455598 |  |  | KX455570 | KC953401 |
| *Microtus kikuchii* | AF163896 |  |  |  | AM392385 |  |
| *Microtus ochrogaster* | DQ432006 | KX455612 |  |  | MF074897 | KX455529 |
| *Microtus pennsylvanicus* | KF948531 | AY295009 | KF948650 | KJ556569 | AF540633 | AM919415 |
| *Myodes andersoni* | AB037281 |  | KJ556809 | AB086033 | AM392391 | KJ556764 |
| *Myodes californicus* | KJ556722 |  | KJ556820 | KJ556608 | MF074901 | KJ556775 |
| *Myodes centralis* | KJ556625 |  | KJ556783 | KJ556573 |  | KJ556738 |
| *Myodes gapperi* | KJ789561 | AY295010 | KJ556806 | KJ556596 | AF540623 | AY326080 |
| *Myodes glareolus* | FJ881411 | JX440346 | KJ556786 | KJ556576 | JF930118 | JX457705 |
| *Myodes regulus* | NC016427 |  |  | AB086037 |  |  |
| *Myodes rex* | AB031582 |  |  | AB086029 |  |  |
| *Myodes rufocanus* | AY309412 | KX455590 | KJ765294 | JF930102 | KX455562 | KX455508 |
| *Myodes rutilus* | AY309424 | KC962247 | KJ765331 | KJ556609 | KC962291 | KJ765262 |
| *Myodes smithii* | AB037304 |  | KJ556805 | AB086036 |  | KJ556759 |

**Table S3** Gene flow of three maternal clades of *E. melanogaster* estimated by Migrate-n. C represents the Central Clade, SE represents the Southeast Clade and SW represents the Southwest Clade.

|  | C to SE | SE to C | C to SW | SW to C | SW to SE | SE to SW |
| --- | --- | --- | --- | --- | --- | --- |
| CYTB | 3.13 | 93.887 | 0.639 | 76.671 | 38.38 | 38.502 |
| BRCA | 4.123 | 51.978 | 6.143 | 45.12 | 18.056 | 144.594 |
| ETS2 | 4.415 | 43.949 | 0.987 | 44.895 | 30.415 | 52.54 |
| G6pd | 8.993 | 13.99 | 1.067 | 13.57 | 13.426 | 5.748 |
| GHR | 4.418 | 55.935 | 0.764 | 58.569 | 40.739 | 31.025 |
| IRBP | 4.613 | 47.376 | 0.804 | 48.348 | 12.663 | 21.151 |
