# Supplementary figures and images for: Disjunct distribution and distinct intraspecific diversification of Eothenomys melanogaster in South China
Source: BMC Evol Biol. 2018 Apr 10;18:50. doi: 10.1186/s12862-018-1168-3 (PMC5894153; doi:10.1186/s12862-018-1168-3)

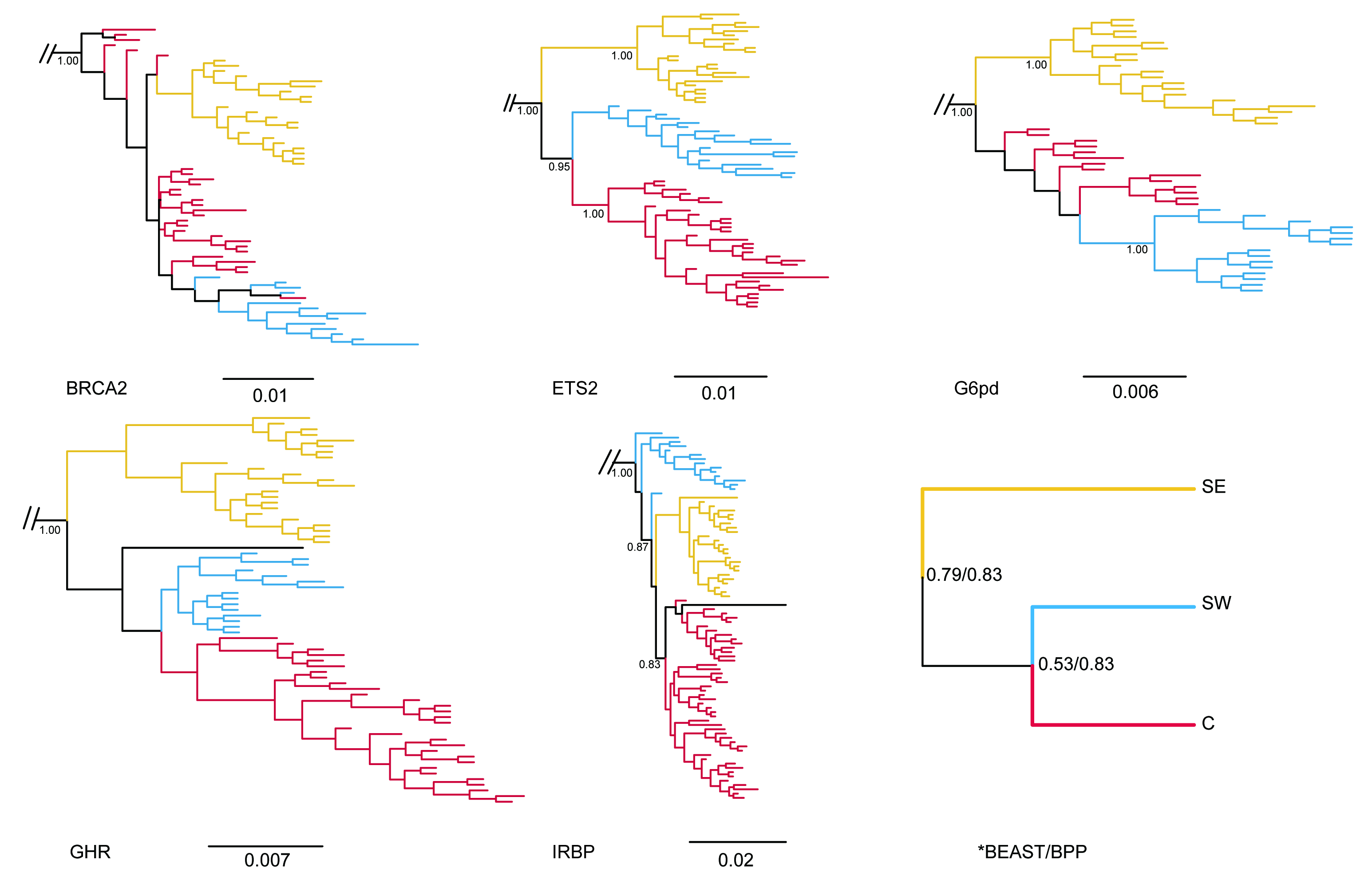

Supplement: Supplementary file 2 — Figure S1. Bayesian phylogenetic trees of nuclear loci and species tree. Bayesian phylogenetic trees of nuclear loci and species tree estimated by BP&P and *BEAST for E. melanogaster. Red indicates individuals belonging to the Central clade; blue indicates individuals belonging to the Southwest clade; and yellow indicates individuals belonging to the Southeast clade. Values beside branches indicate Bayesian posterior probability. (TIFF 24617 kb) [file 12862_2018_1168_MOESM2_ESM.tif]

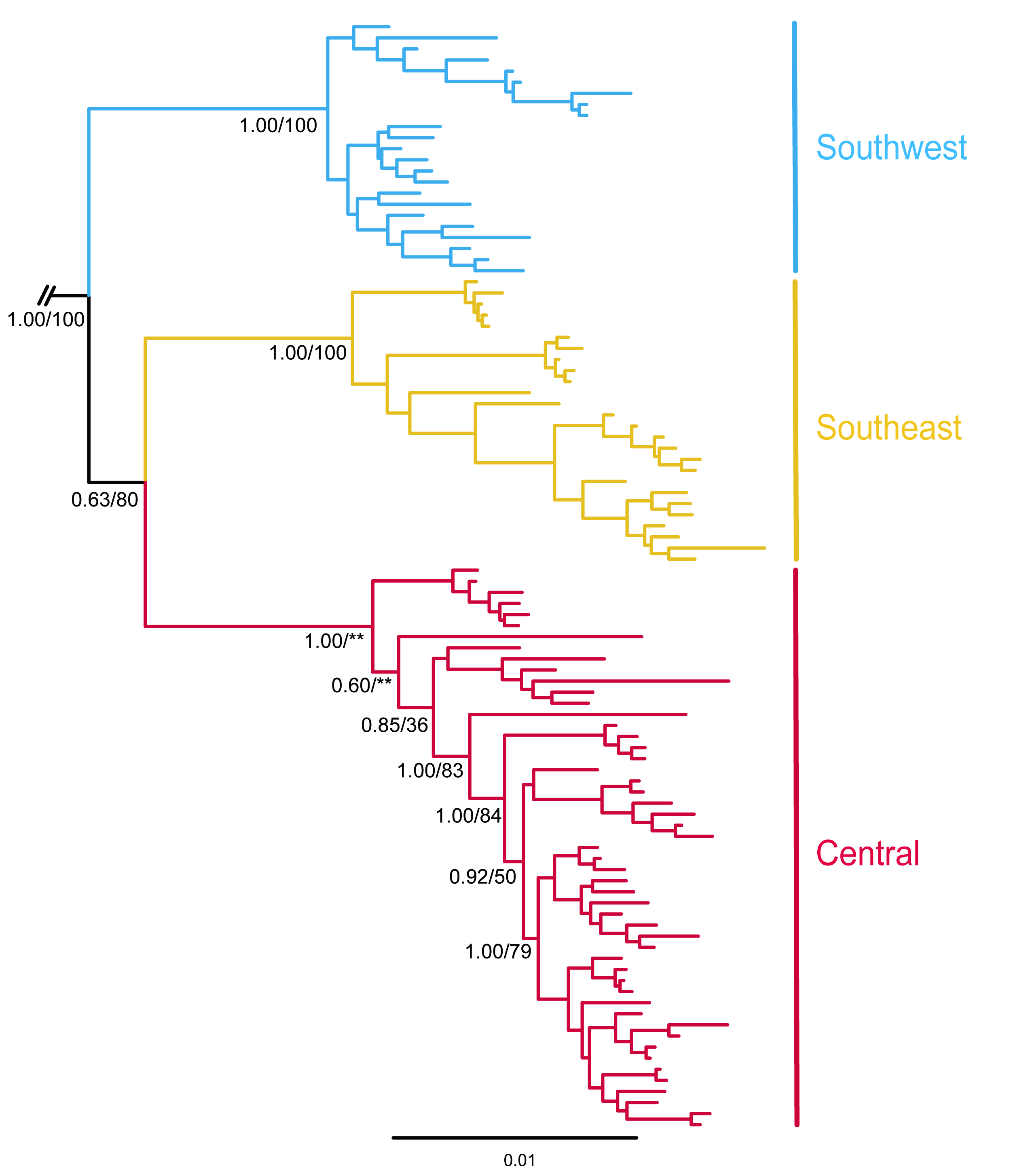

Supplement: Supplementary file 3 — Figure S2. Phylogenetic trees of concatenated cytb and nDNA genes. Phylogenetic trees of concatenated cytb and nDNA genes for E. melanogaster. Values beside branches indicate posterior probability and bootstrap. (TIFF 28922 kb) [file 12862_2018_1168_MOESM3_ESM.tif]
